# Procedural Case: Neonatal Lumbar Puncture

**DOI:** 10.21980/J8.52364

**Published:** 2025-12-31

**Authors:** Charles Lei, Stephanie Cohen, Andrew Melendez, Neil Wallace, Tiffany Moadel, Lars Beattie, Tina Chen, David Fernandez, Stephanie Stapleton, Alaa Aldalati

**Affiliations:** 1 Hennepin County Medical Center, Department of Emergency Medicine, Minneapolis, MN; 2 University of Central Florida, Department of Emergency Medicine, Orlando, FL; 3 Yale University, Department of Emergency Medicine, New Haven, CT; 4 University of Arizona/Banner Medical Center, Department of Emergency Medicine, Tuscon, AZ; 5 Zucker School of Medicine at Hofstra/Northwell, Department of Emergency Medicine, Hempstead, NY; 6 University of Florida, Department of Emergency Medicine, Gainesville, FL; 7 St. Louis University, Department of Emergency Medicine, St. Louis, MO; 8 Mount Sinai Hospital, Department of Emergency Medicine, New York, NY; 9 Boston University/Boston Medical Center, Department of Emergency Medicine, Boston, MA; 10 University of Kansas School of Medicine-Wichita, Wesley Medical Center Emergency Department, Wichita, KS

## Abstract

**Audience:**

This case was specifically designed for senior emergency medicine (EM) resident physicians as a preparatory tool for the American Board of Emergency Medicine (ABEM) Certifying Exam. However, it is applicable for EM residents at all levels of training.

**Introduction:**

Lumbar puncture (LP) is a critical diagnostic procedure in evaluating for central nervous system infections in febrile neonates. Recognizing its significance, ABEM has identified LP as a procedure that is integral to the practice of emergency medicine.^1^ Additionally, ABEM’s new Certifying Exam, launching in 2026, will include LP among the procedural skills that examinees may encounter.^2^ Despite its importance, neonatal LP can be technically challenging, with reported success rates of only 50–60%.^3^ Many EM clinicians hesitate to perform the procedure due to concerns about complications and limited hands-on experience during residency training.^4^ Furthermore, the American Academy of Pediatrics’ updated clinical practice guideline for the evaluation and management of febrile infants^5^ has led to a substantial decline in the number of LPs performed in this patient population,^6^ further reducing opportunities for trainees to gain clinical experience. This simulation case was designed to allow learners to practice and demonstrate the cognitive and technical skills necessary for performing a neonatal LP in a structured, risk-free environment.

**Educational Objectives:**

This is a Procedure case involving a neonatal LP. The overarching educational goal of this case is to assess learners’ clinical decision-making, technical proficiency, and communication skills when performing a neonatal LP. Participants will be evaluated on their ability to identify indications and contraindications, obtain informed consent, prepare for and perform the procedure with sterile technique, and implement appropriate post-procedure care. By the end of the session, learners should be able to: 1) describe the indications and contraindications associated with performing a neonatal LP, 2) obtain informed consent for a neonatal LP, using clear, patient-centered language to explain the procedure and to discuss risks, benefits, and alternative options, 3) demonstrate proper preparation for a neonatal LP, including equipment setup, patient positioning, patient monitoring, use of sterile technique, and analgesia, 4) perform a neonatal LP on a procedural task trainer with technical proficiency, demonstrating proper needle insertion, cerebrospinal (CSF) collection, and adherence to sterile technique, and 5) outline appropriate post-procedure management for the patient, including interpreting CSF results, initiating appropriate treatment, monitoring for complications, and providing caregivers with clear follow-up guidance.

**Educational Methods:**

We developed a single-station Objective Structured Clinical Examination (OSCE) centered on the neonatal LP procedure. This format aligns with the Procedures module of the new ABEM Certifying Exam, which emphasizes real-time procedural performance within simulated clinical encounters. This OSCE station incorporates a neonatal LP procedural task trainer and a standardized examiner script modeled after the ABEM Certifying Exam format to promote realism, consistency, and educational relevance. The case was collaboratively developed by experts in EM and simulation-based education and was externally peer-reviewed to ensure clinical accuracy, scenario clarity, and educational value. During the OSCE, the learner is presented with a brief case summary describing a febrile neonate requiring a lumbar puncture and is expected to demonstrate the essential cognitive and technical skills for performing the procedure. The examiner follows a structured script to deliver standardized prompts, offer procedural cues, and evaluate performance using a detailed, behaviorally anchored checklist that captures both procedural steps and critical decision-making elements.

**Research Methods:**

The simulation case was originally developed by three experts in simulation-based education and EM. To enhance accuracy, realism, and educational value, the case underwent a structured peer review process involving a panel of three additional experts. Reviewers used the Simulation Scenario Evaluation Tool (SSET)^7^ to provide standardized feedback on key elements, including scenario flow, realism, clarity of learning objectives, and alignment with assessment criteria.

The case was subsequently piloted at two academic institutions and a national EM conference to assess its feasibility, clarity, and instructional design in real-world educational settings. During these pilot sessions, faculty facilitators and participating learners interacted with the case using a standardized examiner script and procedural checklist. Feedback and observations from this testing informed refinements to case logistics, examiner prompts, and assessment criteria to enhance usability and educational effectiveness.

**Results:**

Our expert reviewers, using the SSET survey, strongly agreed that the simulation case’s learning objectives were specific, action-oriented, relevant, and appropriately tailored to the target audience’s skill level and knowledge base. They also strongly agreed that the clinical context, embedded critical actions, and patient states effectively supported these learning objectives. Faculty facilitators reported that the scenario materials and resources provided sufficient information to enable independent facilitation of the case at their own institutions. Participating learners indicated that the written materials and verbal instructions were clear and that the experience was valuable for preparing for the ABEM Certifying Exam.

**Discussion:**

This neonatal LP simulation case was effective in achieving its educational objectives and served as a valuable tool for preparing learners for the Procedures module of the ABEM Certifying Exam. Facilitators rated the case highly across key domains, highlighting the clarity, usability, and cohesiveness of the provided materials. Learners consistently reported that the scenario provided meaningful opportunities to practice both procedural skills and clinical reasoning. These findings support the incorporation of structured procedural OSCEs into EM training programs. By leveraging simulation, this case helps address educational gaps, promote consistency in training, and enhance learner preparedness for board certification.

**Topics:**

Lumbar puncture, meningitis, procedure, American Board of Emergency Medicine, Certifying Exam.

## USER GUIDE

**Table:** 

List of Resources:	
Abstract	106
User Guide	109
For Examiner Only	112
Certifying Exam Assessment	117
Stimulus	119
Debriefing and Evaluation Pearls	122

**Learner Audience:**

This case is appropriate for interns and junior and senior residents.

**Time Required for Implementation:**

Case: 10 minutes

Debriefing: 10 minutes

**Recommended number of learners per instructor:**

This case was specifically designed for one senior emergency medicine (EM) resident physician per instructor for individual practice to prepare for the American Board of Emergency Medicine (ABEM) Certifying Exam. However, it is applicable for EM residents at all levels of training.

**Topics:**

Lumbar puncture, meningitis, procedure, American Board of Emergency Medicine, Certifying Exam.

**Objectives:**

By the end of the session, learners should be able to:

1. Describe the indications and contraindications associated with performing a neonatal lumbar puncture (LP)
2. Obtain informed consent for a neonatal LP, using clear, patient-centered language to explain the procedure and to discuss risks, benefits, and alternative options
3. Demonstrate proper preparation for a neonatal LP, including equipment setup, patient positioning, patient monitoring, use of sterile technique, and analgesia
4. Perform a neonatal LP on a procedural task trainer with technical proficiency, demonstrating proper needle insertion, cerebrospinal fluid (CSF) collection, and adherence to sterile technique
5. Outline appropriate post-procedure management for the patient, including interpreting CSF results, initiating appropriate treatment, monitoring for complications, and providing caregivers with clear follow-up guidance

### Linked objectives, methods and results

The primary objective of this simulation case is to familiarize EM residents with the structure and expectations of the Procedures module of the ABEM Certifying Exam while building procedural competence in neonatal lumbar puncture. The OSCE format was purposefully selected for its alignment with the ABEM Certifying Exam, providing a realistic, time-constrained environment that evaluates both procedural skills and clinical reasoning.

This single-station OSCE allows learners to verbalize critical actions and perform the LP procedure on a neonatal task trainer, guided by a standardized examiner script and behaviorally anchored checklist. The structure supports performance at the “shows how” level of Miller’s Pyramid of clinical competence^10^ and integrates principles of deliberate practice,^11^ promoting focused repetition in a high-stakes, lower frequency scenario with immediate, structured feedback.

Each learning objective is intentionally addressed through the OSCE design. The case involves a 14-day-old infant presenting with a fever. The facilitator begins the case by informing the learner that they have decided that a lumbar puncture is indicated for this patient and prompts the learner to address each of the case objectives. The learner should describe the indications (eg, meningitis, encephalitis, neonatal fever) and contraindications (eg, infection at the skin puncture site, concern for coagulopathy or severe thrombocytopenia, concern for elevated intracranial pressure due to mass effect) associated with performing a neonatal LP (Objective 1). The learner should obtain informed consent for a neonatal LP, using clear patient-centered language to explain the procedure to a caregiver. This explanation should include a discussion of the risks, benefits, and alternative options related to the procedure (Objective 2). Next, the learner should demonstrate proper preparation for the procedure. They should gather and set up the necessary supplies, properly position the patient, identify appropriate landmarks, don personal protective equipment and sterile gloves, and administer analgesic therapy (Objective 3). The learner should then perform the LP on a procedural task trainer with technical proficiency. They should demonstrate appropriate execution of each step of the procedure, including correct needle insertion, CSF collection, and adherence to sterile technique (Objective 4). Finally, the facilitator will provide the learner with the results of the CSF analysis. The learner should correctly interpret the CSF results, discuss next steps in treatment, and describe how they would counsel caregivers and monitor for complications (Objective 5).^8^

### Recommended pre-reading for instructor

- Euerle BD. Spinal puncture and cerebrospinal fluid examination. In: Roberts JR, Custalow CB, Thomsen TW, eds. *Roberts and Hedges’ Clinical Procedures in Emergency Medicine and Acute Care*, 7^th^ ed. Elsevier; 2018:1258–1280.^8^
- Auerbach M, Chang T, Krantz A, et al. Infant Lumbar Puncture: POISE Pediatric Procedure Video. *MedEdPORTAL*. 2011;7:8339.
https://doi.org/10.15766/mep_2374-8265.8339

### Results and tips for successful implementation

This simulation case can be implemented either as a standalone scenario or as part of a comprehensive ABEM Certifying Exam practice session. For optimal realism, we recommend that residents complete the case individually. However, it is also suitable for small-group practice across all training levels. Incorporating a mock facilitator role may help residents gain insight into the examiner’s perspective. The scenario is designed to be completed within 10 minutes, with an additional 10 minutes allocated for feedback. To enhance fidelity and support performance assessment, we recommend the use of a commercially available neonatal LP task trainer. Alternatively, facilitators may employ a homemade procedural model,^12^ use an adult LP task trainer, or administer the case in an oral board format with learners verbalizing each procedural step. We tested the case using an iterative trialing process with a convenience sample of EM residents. Faculty facilitators and resident participants provided feedback via anonymous surveys that included 5-point Likert scale items (1 = strongly disagree, 5 = strongly agree) and open-ended response questions. Data were collected using Qualtrics (https://www.qualtrics.com) and analyzed using Excel (Microsoft, Redmond, WA). The project was reviewed and deemed exempt by the Boston University Institutional Review Board.

In the initial round of trialing, a facilitator at an academic institution tested the case with two EM residents. The facilitator evaluated the simulation using the Simulation Scenario Evaluation Tool (SSET)^7^ while learners completed a modified usability survey. We conducted a second round of testing at the Society for Academic Emergency Medicine Annual Meeting in May 2025 (Philadelphia, PA), followed by a third round at a separate academic institution with an EM residency program. In these latter rounds, two facilitators and six EM residents completed modified usability surveys. We revised the case after each round based on survey feedback.

Feedback from the SSET was predominantly positive. The facilitator in the initial round strongly agreed that the simulation case’s learning objectives were specific, action-oriented, relevant, and appropriately tailored to the target audience’s skill level and knowledge base. They also strongly agreed that the clinical context, critical actions, and patient states were clearly defined and effectively supported the learning objectives. In the second and third rounds, facilitators (n = 2) rated the case highly across all domains, with mean scores of 4.0 out of 5 for ease of use, material integration, user confidence, and relevance for ABEM Certifying Exam practice. They agreed that the case objectives and materials were clear and expressed interest in continued use of the case for exam preparation.

Resident feedback (n = 6) was similarly positive. Written and verbal materials were consistently rated as clear, with mean scores of 4.5 and 4.3, respectively. All participants agreed or strongly agreed that the case was helpful for preparing for the ABEM Certifying Exam. Comments emphasized the case’s usefulness in practicing procedural skills and clinical decision-making under exam-like conditions. Suggestions for improvement incorporated into the final version of the case included clarifying the language around informed consent, standardizing examiner prompts, and accommodating variations in practice (eg, patient positioning, analgesic selection).

## Figures and Tables

**Table:** 

**Candidate Task Sheet**	
**#1**	**Cerebrospinal Fluid Analysis**

**Table:** Procedural Case Candidate Task Sheet

**CASE PARAMETERS**
This is a 10-minute case. You will interact with an examiner and procedural equipment in a simulated scenario. A 14-day-old patient presents to the Emergency Department with a fever. You have decided that a lumbar puncture is indicated for this patient. The model and needed supplies will be available for you on the table.
**PHYSICAL EXAM FINDINGS**
The patient has a temperature of 38.5°C. The remainder of the physical examination is unremarkable.
**TASK STATEMENT**
Your tasks are as follows: Describe the indications and contraindications associated with performing a neonatal lumbar puncture Obtain informed consent for the procedure Describe how you prepare to perform the procedure Perform a lumbar puncture Make recommendations for management based on cerebrospinal fluid (CSF) interpretation

**STIMULUS 1 t4-jetem-10-5-ce106:** CEREBROSPINAL FLUID ANALYSIS

Type	Patient Results	Normal Values
CSF Appearance	Turbid	Clear
White Blood Cell Count	>3000 /μL	<30 /μL
Predominant Cell Type	PMN	Lymphocytes
Red Blood Cell Count	2 /μL	0 /μL
Protein	250 mg/dL	<170 mg/dL
Glucose	8 mg/dL	>30 mg/dL

**Table:** How to interpret CSF studies:^15^

	NORMAL	BACTERIAL	VIRAL	FUNGAL/TB
Appearance	Normal	Turbid	Clear	Fibrin Web
Protein (g/L)	0.18–0.45	>1	<1	0.1–0.5
Glucose (mmol/L)	2.5–3.5	< 2.2	Normal	1.6–2.5
Gram Stain	Normal	60–90% Positive	Normal	
White blood cells	<9.2	>500	<1000	100–500
Other		90% PMN (Polymorpho-nuclear Leukocytes)	Monocytes	Monocytes

## FOR EXAMINER ONLY

### Procedural Case: Neonatal Lumbar Puncture Summary

**Case Summary:** A 14-day old infant presents to the Emergency Department (ED) with a fever. The ED team has decided that a lumbar puncture (LP) is indicated for this patient. The candidate should describe the indications and contraindications of this procedure and demonstrate how to obtain informed consent for the procedure. The candidate should demonstrate how to prepare for and then perform a lumbar puncture on a neonatal lumbar puncture task trainer. The candidate will be provided with the cerebrospinal fluid (CSF) analysis results and should make recommendations for management based on their interpretation of the results.

**Materials/personnel needed:**

Hospital crib

Neonatal LP task trainer

Antiseptic solution

Sterile gloves

Sterile drapes

Analgesic

25- or 22-gauge LP needle

CSF collection tubes

**Room Setup:**

Standard ED room

### Procedural Case: Neonatal Lumbar Puncture Objectives

1. **Case Objective 1:** Describe the indications and contraindications associated with performing a neonatal lumbar puncture (LP). Learners will successfully complete this objective by listing at least two indications (eg, meningitis, encephalitis, neonatal fever) and contraindications (eg, infection at the skin puncture site, concern for coagulopathy or severe thrombocytopenia, concern for elevated intracranial pressure due to mass effect) related to neonatal LP.
2. **Case Objective 2:** Obtain informed consent for a neonatal LP from a caregiver. Learners will successfully complete this objective by using clear, patient-centered language to explain the procedure and to discuss risks, benefits, and alternative options.
3. **Case Objective 3:** Demonstrate proper preparation for a neonatal lumbar puncture. Learners will successfully complete this objective by gathering and setting up the necessary supplies, properly positioning the patient, identifying appropriate landmarks, donning personal protective equipment and sterile gloves, and administering analgesic therapy.
4. **Case Objective 4:** Perform a neonatal lumbar puncture on a procedural task trainer with technical proficiency. Learners will successfully complete this objective by demonstrating appropriate execution of each step of the procedure, including correct needle insertion, cerebrospinal fluid (CSF) collection, and adherence to sterile technique.
5. **Case Objective 5:** Outline appropriate post-procedure management for the patient. Learners will successfully complete this objective by correctly interpreting the CSF results, discussing next steps in treatment, and describing how they would counsel caregivers and monitor for complications.

### Procedural Case: Neonatal Lumbar Puncture Examiner Script

**Examiner:**

Hello, my name is ________. I will be your Examiner for this station. Please have a seat.

This is a Procedure case. I will provide you with some questions and prompts to guide you through this case. You will have 10 minutes to work through the case.

As you saw on the door sign, you are taking care of a 14-day-old newborn with a fever. You have decided that a lumbar puncture is indicated for this patient.

Before we get to the procedure, can you start by describing the indications and contraindications associated with performing a neonatal lumbar puncture?

**Learner:** Response

**Examiner:** You have determined that there are no contraindications to performing the lumbar puncture. Can you describe the process by which you would obtain informed consent?

**Learner:** Response

**Examiner:**

*If the Learner does not mention specific risks and benefits:* What are the risks and benefits specific to a neonatal lumbar puncture that you would discuss with the patient’s caregiver?

*Potential follow-up questions based on the Learner’s response:*

The patient’s caregiver has expressed that they are concerned about potential spinal cord or nerve injury. How would you address their concern?

*If the Learner has answered the above prompt:*

After having that informed conversation with the caregiver, the caregiver is agreeable to doing the procedure and the informed consent has been obtained. Can you describe the steps that you would take to prepare for the lumbar puncture?

**Learner:** Response

**Examiner:**

*Potential follow-up questions based on the Learner’s response:*

What supplies would you gather?

Can you demonstrate and/or describe in detail how you would position and hold the patient for the lumbar puncture?

How would you maintain sterile technique?

*If the Learner has answered the above prompt:*

Okay, your supplies have been gathered to perform the lumbar puncture. You have positioned the patient. Next, we are going to transition to our procedural task trainer. All of the supplies you will need are here. Before you begin the lumbar puncture, I would like to orient you to our task trainer. This is a neonatal lumbar puncture task trainer. This area represents the baby’s head, while that area represents the baby’s feet.

Before we prep the patient, can you describe, on this model, how you would identify your landmarks for this procedure?

**Learner:** Response

**Examiner:** Can you describe how you would provide analgesia?

**Learner:** Response

**Examiner:** Please assume the patient has received analgesic therapy as you have described. How would you prep and drape this patient?

**Learner:** Response

**Examiner:** The patient is now prepped and draped. Now you can perform the lumbar puncture. As you proceed, please verbalize each step of the procedure. There are gloves here for you to use. Please obtain a CSF fluid specimen.

**Learner:** Performs procedure, verbalizing each step

**Examiner:**

*May provide prompts to move procedure forward:*

You have been able to successfully obtain a CSF sample and collect 4 vials of CSF.

*Show the Learner the fluid-filled CSF collection tubes:*

This is what the fluid looks like. Continue with the next steps of the procedure.

**Learner:** Completes procedure

**Examiner:** You can remove your gloves. We are done with the technical portion of the procedure. Please have a seat and we will transition to the next portion. You indicated you would send off the CSF for laboratory analysis. Can you tell me specifically what you would like to order?

**Learner:** Response

**Examiner:** The studies have been obtained. Here are the results of the CSF analysis. Please interpret the CSF results and then provide a diagnosis and recommendation for the next steps in management. Let me know when you are ready to proceed.

**Learner:** Response

**Examiner:** These interpretations and management recommendations have been communicated with the patient’s caregiver, and the patient has been admitted to the hospital. This concludes the Procedure case. Thank you.

## CERTIFYING EXAM ASSESSMENT

### Procedural Case: Neonatal Lumbar Puncture

Learner: _________________________________________

□ List at least two indications and two contraindications associated with performing a neonatal lumbar puncture (LP)
  - Indications: meningitis, encephalitis, neonatal fever
  - Contraindications: infection at the skin puncture site, concern for coagulopathy or severe thrombocytopenia, concern for elevated intracranial pressure due to mass effect
□ Use plain language to explain the procedure to a caregiver
□ List at least two risks associated with performing a neonatal LP
  - Risks: infection, bleeding, damage or irritation of surrounding structures (eg, nerves), pain
□ Gather all necessary supplies
  - Antiseptic solution
  - Sterile gloves
  - Sterile drapes
  - Analgesic
  - 25- or 22-gauge LP needle
  - Cerebrospinal fluid (CSF) collection tubes
□ Perform appropriate hand hygiene and don personal protective equipment
□ Initiate cardiorespiratory and oxygen saturation monitoring
□ Position the neonate
  - Place on a firm surface
  - Place in lateral decubitus or sitting position
  - Flex the lower back
  - Align the spine parallel (lateral decubitus) or perpendicular (sitting) to the bed
□ Palpate for anatomical landmarks and identify the L4–L5 or L5–S1 interspace
□ Clean the skin with antiseptic solution and prepare a sterile field
□ Administer analgesic therapy
□ Insert the spinal needle with proper technique
  - Orient the bevel parallel to the spine
  - Direct the needle perpendicular to the midline of the spine
  - Advance needle incrementally and assess for CSF flow
□ Collect CSF in 4 collection tubes
□ Remove the needle safely and apply a dressing
□ Label the specimens correctly and send for analysis
  - Cell count and differential
  - Protein and glucose
  - Gram stain and culture
  - Herpes simplex virus (HSV) polymerase chain reaction (PCR)
□ Interpret the CSF results and diagnose bacterial meningitis
□ Initiate appropriate intravenous antibiotics for treatment of bacterial meningitis
□ Assess patient post-procedure for any complications

**Summative and formative comments:**

### Stimulus Inventory

[Table t4-jetem-10-5-ce106]

## DEBRIEFING AND EVALUATION PEARLS

### Procedural Case: Neonatal Lumbar Puncture

- Reactions Phase
  *Goal: Allow the learner to process emotional and cognitive responses.*
  - “How did you feel about the case?”
  - “What went well? What did not go well?”
  - “What surprised you during the simulation?”
- Description Phase
  *Goal: Establish a shared understanding of what happened.*
  - “In 2–3 sentences, please summarize this procedural case.”
  - “What helped or hindered your ability to perform the procedure?”
- Analysis Phase
  *Goal: Facilitate reflection and provide structured feedback based on rubric scores.*
  - “What key indications and contraindications for lumbar puncture (LP) guided your decision-making?”
  - “What was your strategy for identifying the correct anatomical landmarks?”
  - “Did the procedure go smoothly? What would you do differently if you encountered this case again?”
- Application/Summary Phase
  - “What is one key thing you learned today that you will apply the next time you perform an LP?”

**Neonatal Lumbar Puncture Clinical Pearls** **13**

Indications:

- Suspected central nervous system (CNS) infection (eg, meningitis, encephalitis)
- Neonatal fever

Contraindications:

- Concern for elevated intracranial pressure (ICP) due to mass effect (eg, tumor, abscess, hemorrhage)
- Coagulopathy
- Severe thrombocytopenia
- Infection at skin puncture site

When to perform head imaging before an LP (potential causes of elevated ICP):

- Prior CNS disease or seizures
- Abnormal immunity
- Abnormal level of consciousness or neurologic exam
- Papilledema

Technique:

- Gather all necessary supplies
  ○ Antiseptic solution
  ○ Sterile gloves
  ○ Sterile drapes
  ○ Anesthetic (eg, topical lidocaine cream, injectable lidocaine for local infiltration, oral sucrose)
  ○ 25- or 22-gauge LP needle
  ○ Cerebrospinal fluid (CSF) collection tubes
- Perform appropriate hand hygiene and don personal protective equipment
- Initiate cardiorespiratory and oxygen saturation monitoring
- Position the patient**13**
  ○ Place patient on firm surface
  ○ Sitting upright: easier to identify midline; flex lower back; spine perpendicular to bed
  ○ Lateral recumbent: flex hips/knees to chest and arch lower back; spine parallel to bed; line drawn between shoulders and line drawn between hips perpendicular to bed; back perpendicular to bed
- Palpate for anatomical landmarks
  ○ Palpate iliac crests to identify L4 spinous process
  ○ Identify L4–L5 and L5–S1 interspaces
- Clean the skin with antiseptic solution and prepare a sterile field
- Administer analgesic therapy
- Insert the spinal needle with proper technique
  ○ Orient needle with bevel facing up if the patient is in the lateral recumbent position and to the side if the patient is sitting upright (parallel to the spine)
  ○ Direct needle perpendicular to midline of spine
  ○ Advance needle and stylet as a unit, periodically removing stylet during incremental advancement to assess for CSF flow
  ○ Alternatively, remove stylet after skin transection and advance needle until CSF return is visualized**14**
  ○ If bone is encountered, partially remove needle and redirect cephalad or caudad
- Collect CSF
  ○ Sequentially fill 4 tubes (approximately 1–2 mL per tube)
- Replace stylet, completely withdraw needle, hold pressure at entry site, and place bandage

Post-Procedure:

- Label tubes and order CSF studies
  ○ Cell count with differential (use least bloody tube based on visual inspection)
  ○ Protein and glucose
  ○ Gram stain and culture
  ○ Herpes simplex virus (HSV) polymerase chain reaction (PCR)

Complications:

- Infection
- Bleeding
- Herniation
